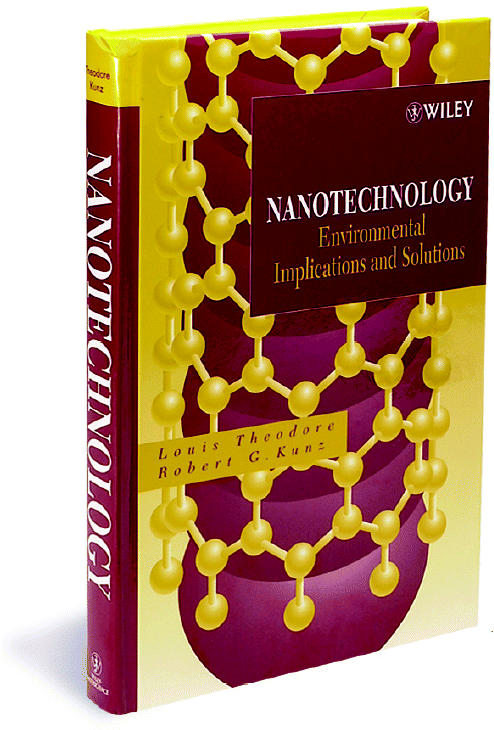# Nanotechnology: Environmental Implications and Solutions

**Published:** 2005-07

**Authors:** Eva Oberdörster

**Affiliations:** Eva Oberdörster earned her Ph.D. at Duke University and completed her postdoctoral work at Tulane University. She is currently on the faculty at Southern Methodist University in the Department of Biology, where her most recent research focuses on ecotoxicology of engineered nanoparticles.

By Louis Theodore and Robert G. Kunz

Hoboken, NJ:John Wiley & Sons, 2005. 378 pp. ISBN: 0-471-69976-4, $99.95 cloth

This book, written mainly for engineering students, gives an excellent summary of traditional environmental pollution issues. Ten chapters cover current legislation regarding environmental pollutants; an overview of chemistry and current nanotechnology processes; air, water and solid-waste issues; multimedia analysis; both health and hazard risk assessment; ethical considerations; and concluding remarks on future trends.

Nanotechnology is emerging in a wide variety of applications, yet unfortunately very little is known about the environmental implications of engineered nanomaterials, of possible ways to handle engineered nano-materials as environmental pollutants, or of how nanomaterials behave in the environment when used for remediation. Therefore, such a book will increase awareness of potential problems, but may disappoint those who expect dramatic revelations about nanoparticles as pollutants.

Due to the current paucity of data, the book gives a well-written overview of environmental toxicology of traditional toxicants, with only a bare mention of nanomaterials in introductions to each chapter. The exception is Chapter 2, which covers basic chemistry and various processes used in making nanosized materials. A few analytical tools currently used to study nanomaterials are discussed, followed by a good overview of the current and near-future uses of nanotechnology. Other chapters present traditional monitoring methods and pollution control methods in great detail, and are useful for teaching. However, the authors do not delve into the current literature showing that nanosized materials do not necessarily behave like bulk materials in water and soils and that it would be prudent to consider and develop new treatment or containments strategies.

The long section on current pollution-related regulations implies that laws already exist that apply to engineered nanomaterials. However, the book does not mention several recent rulings stating that the nano-sized materials are not considered different from bulk (until or unless they receive their own Chemical Abstracts Service numbers), and therefore most of the legislation does not apply to these materials. Although Occupational Safety and Health Administration legislation is mentioned, the impressive ongoing effort by scientists from the National Institute for Occupational Safety and Health to develop monitoring tools and toxicity data for nanosized materials is absent from this section.

Because much of the information currently known about nano-sized particles comes from air pollution and inhalation toxicology, the section on air pollution is more robust than other chapters in terms of nano-implications. But the section contains some inaccuracies. The authors assume that nanosized materials behave as gases—which is true only for very small particles (~ < 5 nm). When particle concentrations increase, they can quickly aggregate and behave like larger-sized particles. There is bare mention of the importance of particle chemistry, surface chemistry, concentration, shape, and size in nanoparticles or engineered nanomaterial behavior as air pollutants. Other inaccuracies include the statement that there is no information on deposition of nanosized particles in the respiratory tract, and repeated misuse of terms such as “particulate” versus “particle.” However, these inaccuracies do not distract from the otherwise thorough discussions of pollution issues that introduce the interested engineer to ecotoxicology. The water and solid-waste sections of the book focus on traditional toxicants as case studies and give a detailed description of current waste-water and solid-waste treatment. With few toxicity or exposure data available, the risk assessment and hazard assessment sections focus on traditional toxicants as case studies—a good foundation for future engineers dealing with the emerging risks and hazards of nanotechnology. A chapter on ethics presents several fictional scenarios, useful in classroom discussions on environmental ethics and the role of environmental engineers in monitoring and responsible whistle blowing.

Overall, the book’s good overview of traditional (bulk) toxicants serves as background for potential nanosized material problems. Given the scarcity of data about nanoecotoxicology, very little information now exists on environmental implications of nanotechnology. Such information should become available over the next 5 years, contributing to a second edition of this book.

## Figures and Tables

**Figure:**